# Supplementary material for: Evolutionarily Developed Alternatively Spliced Exons Containing Translation Initiation Sites
Source: Cells. 2024 Dec 26;14(1):11. doi: 10.3390/cells14010011 (PMC11719525; doi:10.3390/cells14010011)
Supplement: Supplementary file 1 [file cells-14-00011-s001.zip › Figure S1.pdf]

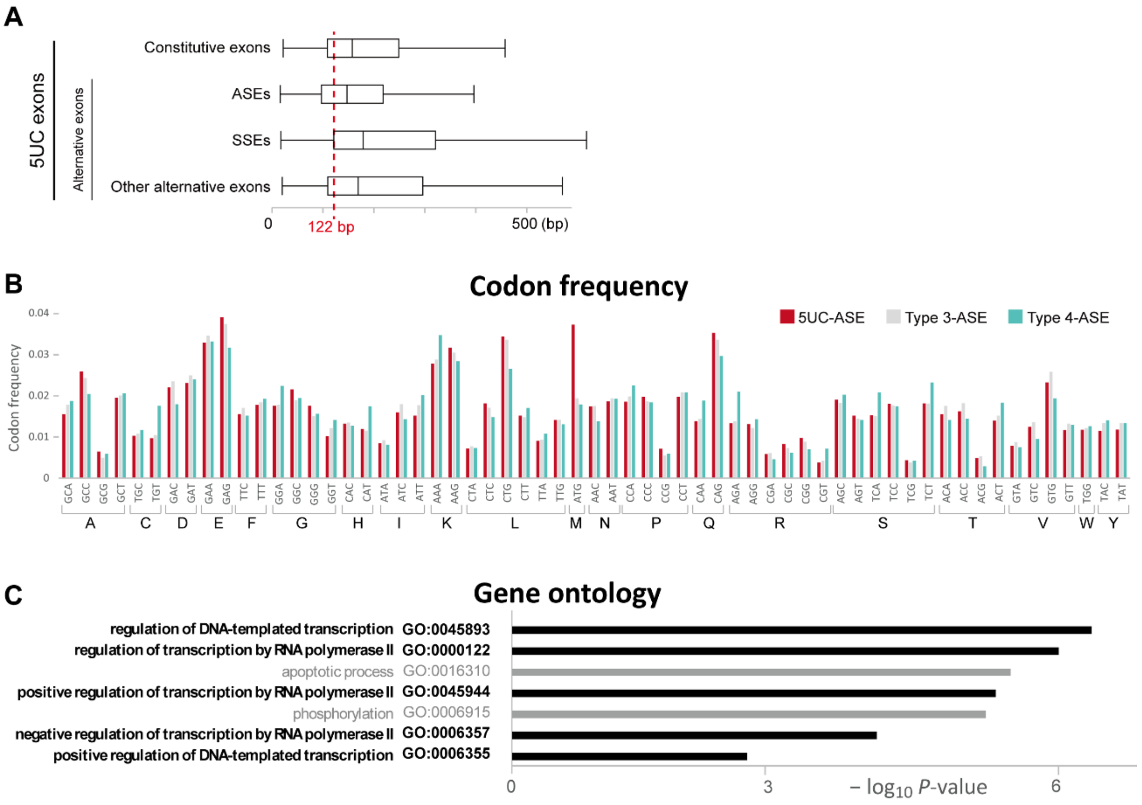

Figure S1. In contrast to Figure 1, where constitutive exons were analyzed, the analysis of alternative exons is shown here. (**A**) Boxplot showing the exon lengths of constitutively spliced 5UC exons and alternatively spliced 5UC exons (ASEs, SSEs, and other alternative exons). (**B**) Bar graph showing the codon usage of 5UC-ASEs, Type 3-ASEs, and Type 4-ASEs. (**C**) Bar graph showing the enriched gene ontology (GO) terms of 5UC-ASE genes. The GO terms associated with DNA binding and transcriptional regulation are indicated in black.
