# Supplementary material for: Evolutionarily Developed Alternatively Spliced Exons Containing Translation Initiation Sites
Source: Cells. 2024 Dec 26;14(1):11. doi: 10.3390/cells14010011 (PMC11719525; doi:10.3390/cells14010011)
Supplement: Supplementary file 1 [file cells-14-00011-s001.zip › Figure S2.pdf]

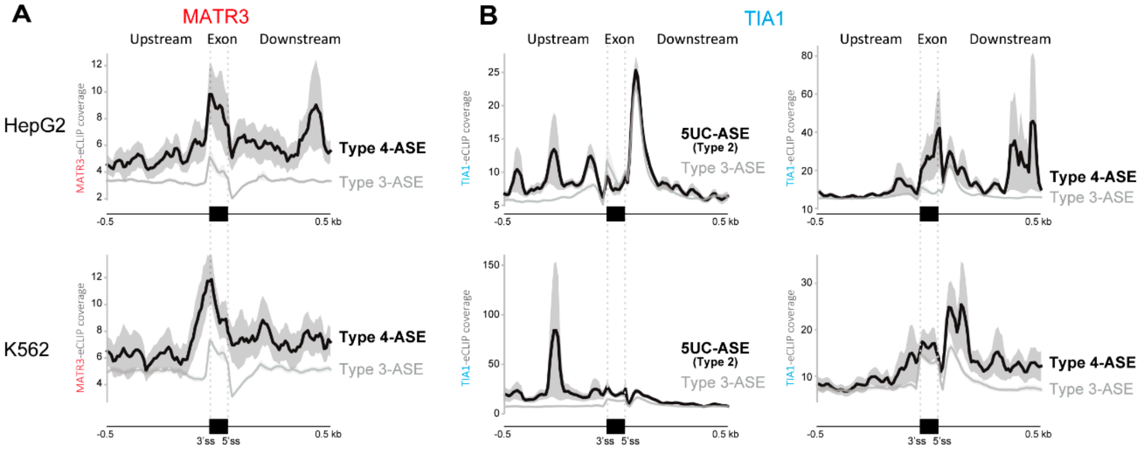

Figure S2. (**A**) Profile plots showing the distribution of MATR3-RNA interactions from 500 nt upstream to 500 nt downstream of Type 4-ASEs (black) and Type 3-ASEs (gray) in HepG2 and K562 cells. In contrast to Figure 3A, where 5UC-ASEs were analyzed, Type 4-ASEs were analyzed here. (**B**) Profile plots showing the distribution of TIA1-RNA interactions from 500 nt upstream to 500 nt downstream of 5UC-ASEs (left) and Type 4-ASEs (right) in HepG2 and K562 cells. In contrast to Figure 3A, where MATR3 was analyzed, TIA1 was analyzed here. The standard error of the average coverage of eCLIP reads is shown as a gray shade around the average curve.
