# Supplementary material for: Evolutionarily Developed Alternatively Spliced Exons Containing Translation Initiation Sites
Source: Cells. 2024 Dec 26;14(1):11. doi: 10.3390/cells14010011 (PMC11719525; doi:10.3390/cells14010011)
Supplement: Supplementary file 1 [file cells-14-00011-s001.zip › Figure S3.pdf]

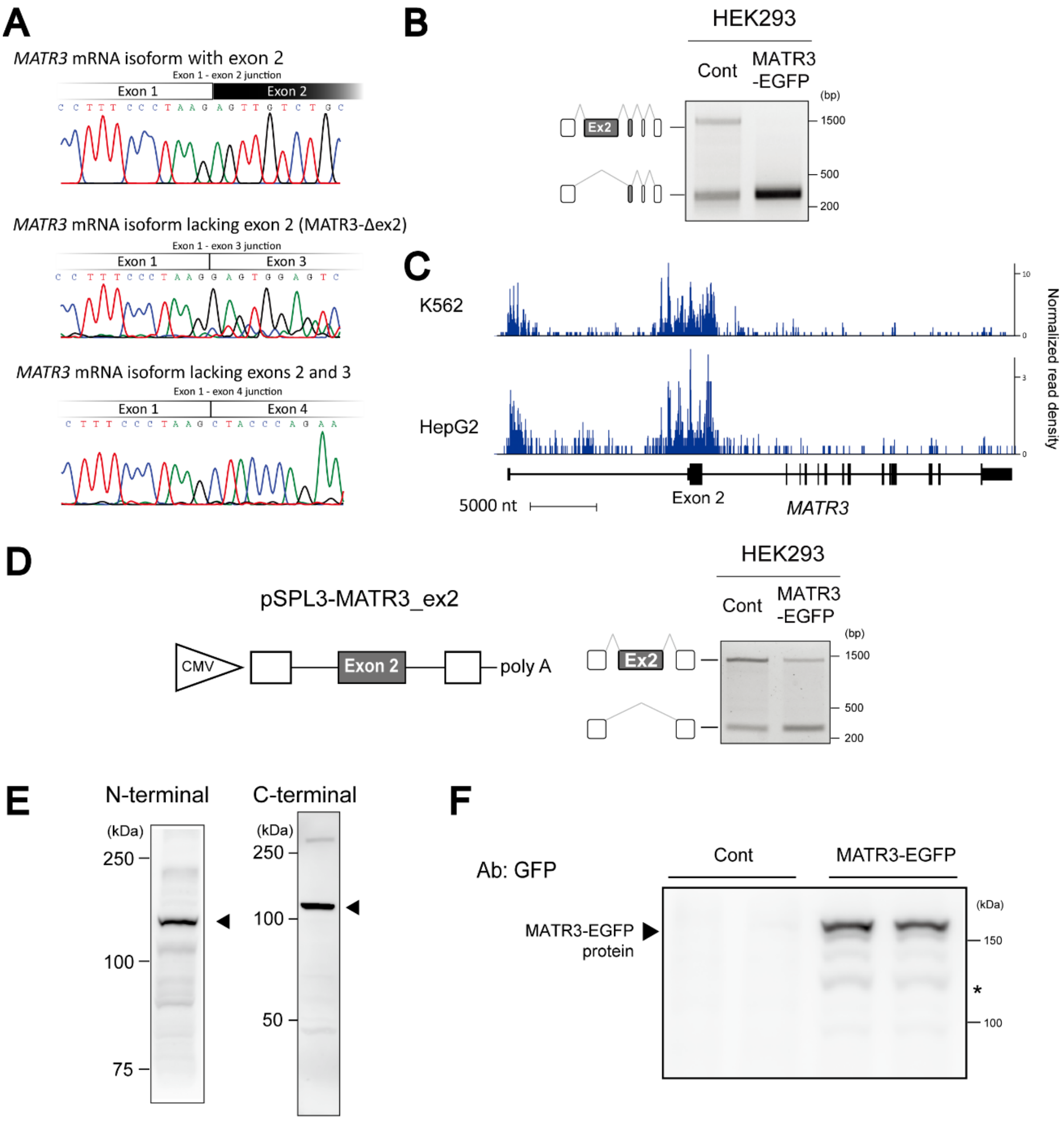

Figure S3. (A) Sanger sequencing analysis showing the skipping of exon 2 and exon 3 in the PCR products shown in Figure 4A. **(B)** Overexpression of MATR3-EGFP promotes skipping of *MATR3* exon 2 of endogenous *MATR3* mRNA. HEK293 cells were transfected with MATR3-EGFP or control (EGFP alone: Cont) expression vectors. RT-PCR analysis was performed 48 hours after the transfection using the primer sets specific to endogenous *MATR3* mRNA. **(C)** Distribution of MATR3 bindings on *MATR3* detected in MATR3 eCLIP-seqs using K562 and HepG2 cells. **(D)** Overexpression of MATR3-EGFP promotes the skipping of exon 2 in the transcript of *MATR3* minigene (pSPL3-MATR3_ex2). HEK293 cells were transfected with MATR3-EGFP or control (EGFP alone: Cont) expression vectors along with pSPL3-MATR3_ex2 minigene. RT-PCR analysis was performed 48 hours after the transfection using the primer set specific to the minigene. Schematic representation of pSPL3-MATR3_ex2 is shown on the left. **(E)** Western blot showing expression of MATR3 protein isoforms. Whole-cell lysates were harvested from HEK293 cells and Western blotting was performed with anti-N-terminal or anti-C-terminal MATR3 antibody. **(F)** Western blot showing expression of MATR3-EGFP protein in HEK293 cells overexpressing MATR3-EGFP. The cell lysates used in Figure 4E were analyzed. Western blotting was performed with anti-GFP antibody. The asterisk denotes fragmented MATR3-EGFP protein that were also detected in the blot with anti-MATR3 antibody (Figure 4E).
