# Supplementary material for: Evolutionarily Developed Alternatively Spliced Exons Containing Translation Initiation Sites
Source: Cells. 2024 Dec 26;14(1):11. doi: 10.3390/cells14010011 (PMC11719525; doi:10.3390/cells14010011)
Supplement: Supplementary file 1 [file cells-14-00011-s001.zip › Table S1.pdf]

Table S1. Accession numbers of eCLIP bam format files of 33 splicing factors registered in ENCODE.

Splicing factor	HepG2		K562	
AQR	ENCFF882PEQ	ENCFF947JHV	ENCFF374DAW	ENCFF436KLO
BUD13	ENCFF536HGU	ENCFF644DWW	ENCFF563BNB	ENCFF086LQG
EFTUD2	ENCFF499JDZ	ENCFF061QCD	ENCFF187FER	ENCFF703QKL
FUS	ENCFF177YJA	ENCFF181DXK	ENCFF210ZRQ	ENCFF341YQ
FXR2	ENCFF157JBC	ENCFF500VO	ENCFF301ANL	ENCFF272ZMQ
HNRNPA1	ENCFF330OFU	ENCFF690NVV	ENCFF571IRN	ENCFF876SGL
HNRNPC	ENCFF630YNF	ENCFF162FHQ	ENCFF048BIQ	ENCFF935AZ
HNRNPK	ENCFF457EXY	ENCFF329QRR	ENCFF704FID	ENCFF570EJV
HNRNPL	ENCFF518JBW	ENCFF607WID	ENCFF775AIE	ENCFF993XXG
HNRNPM	ENCFF950RRY	ENCFF633FLY	ENCFF050PTL	ENCFF379LZD
HNRNPU	ENCFF587PLY	ENCFF125EP	ENCFF025YVA	ENCFF197OBL
KHSRP	ENCFF961WNO	ENCFF701PJM	ENCFF698TGL	ENCFF394THC
LARP7	ENCFF345DPL	ENCFF086NCN	ENCFF863HRM	ENCFF476VSU
MATR3	ENCFF619UHD	ENCFF601NWD	ENCFF162SAS	ENCFF014KBZ
NCBP2	ENCFF138FXS	ENCFF025VQL	ENCFF913QVA	ENCFF308LKS
PRPF8	ENCFF205BLM	ENCFF672HF	ENCFF987VD	ENCFF558FAR
PTBP1	ENCFF295MQP	ENCFF386UI	ENCFF765BPN	ENCFF659RKW
QKI	ENCFF567ADV	ENCFF862YVK	ENCFF698BKX	ENCFF012WMS
RBFOX2	ENCFF994WPX	ENCFF154BQS	ENCFF537RYR	ENCFF296GDR
RBM15	ENCFF562QV	ENCFF261SEG	ENCFF739LLZ	ENCFF086HIT
RBM22	ENCFF051SNH	ENCFF169EKN	ENCFF045LAO	ENCFF391IBH
SF3B4	ENCFF040BYE	ENCFF483VLR	ENCFF095KWC	ENCFF173BOO
SMNDC1	ENCFF110YDG	ENCFF125BMO	ENCFF253SPF	ENCFF770DEI
SRSF1	ENCFF867TIG	ENCFF418ZXO	ENCFF810FCS	ENCFF305QSS
SRSF7	ENCFF743ZXA	ENCFF558CNE	ENCFF149XNS	ENCFF937IVH
SRSF9	ENCFF651VGM	ENCFF460TAE	ENCFF870JPN	ENCFF937IDV
TAF15	ENCFF420FTB	ENCFF701ZSE	ENCFF501PCN	ENCFF044RGH
TARDBP	ENCFF918MZ	ENCFF792QOH	ENCFF862SNV	ENCFF748KMS
TIA1	ENCFF258DPQ	ENCFF681JJG	ENCFF080VML	ENCFF581EBE
TRA2A	ENCFF745EHU	ENCFF372BZV	ENCFF159MVB	ENCFF846XXE
U2AF1	ENCFF755DUB	ENCFF059KEP	ENCFF314SWS	ENCFF132PVT
U2AF2	ENCFF358STL	ENCFF033XVX	ENCFF835KXL	ENCFF936JSP
UPF1	ENCFF811KHK	ENCFF699EEF	ENCFF948AOR	ENCFF701TPG
