# Supplementary material for: Evolutionarily Developed Alternatively Spliced Exons Containing Translation Initiation Sites
Source: Cells. 2024 Dec 26;14(1):11. doi: 10.3390/cells14010011 (PMC11719525; doi:10.3390/cells14010011)
Supplement: Supplementary file 1 [file cells-14-00011-s001.zip › Table S3.pdf]

Table S3. Accession numbers of the transcript quantification files derived from RNA-seq of *MATR3*/*TIA1* silenced and control cells registered in ENCODE. (A) RNA-seqs of HepG2 cells and (B) those of K562 cells.

A. HepG2 cells.

MATR3		TIA1	
**shRNA-seq**	**control RNA-seq**	**shRNA-seq**	**control RNA-seq**
ENCFF365FUM	ENCFF316ORK	ENCFF332UXY	ENCFF700ZBE
ENCFF090YAE	ENCFF017SPB	ENCFF787GVZ	ENCFF816GAS

B. K562 cells.

MATR3		TIA1	
**shRNA-seq**	**control RNA-seq**	**shRNA-seq**	**control RNA-seq**
ENCFF174HBZ	ENCFF875PEA	ENCFF524HXS	ENCFF875PEA*
ENCFF281ZBQ	ENCFF180IOW	ENCFF804IVU	ENCFF180IOW*

*Same as the control files in the MATR3 analysis of K562 cells due to the lack of K562 cell control files for TIA1 in ENCODE.
