# Supplementary material for: Evolutionarily Developed Alternatively Spliced Exons Containing Translation Initiation Sites
Source: Cells. 2024 Dec 26;14(1):11. doi: 10.3390/cells14010011 (PMC11719525; doi:10.3390/cells14010011)
Supplement: Supplementary file 1 [file cells-14-00011-s001.zip › Table S4.pdf]

Table S4. GO terms associated with 5UC-ASE genes in human, rhesus macaque, cow, mouse, rat, chicken, frog, zebrafish, medaka, and elephant shark. The GO terms associated with at least 5% of the input genes were extracted from the results of DAVID's GO BP DIRECT.

Species	GO ID	GO term	Count	%	*P*-value
**Human**	**GO:0006355**	**regulation of DNA-templated transcription**	**82**	**7.211961302**	**4.27E-07**
	**GO:0006357**	**regulation of transcription by RNA polymerase II**	**131**	**11.52154793**	**9.76E-07**
	GO:0006915	apoptotic process	60	5.277044855	3.31E-06
	**GO:0045944**	**positive regulation of transcription by RNA polymerase II**	**105**	**9.234828496**	**4.81E-06**
	GO:0016310	phosphorylation	62	5.45294635	6.21E-06
	**GO:0000122**	**negative regulation of transcription by RNA polymerase II**	**80**	**7.036059807**	**9.78E-05**
	**GO:0045893**	**positive regulation of DNA-templated transcription**	**57**	**5.013192612**	**0.002605249**
**Rhesus macaque**	**GO:0006355**	**regulation of DNA-templated transcription**	**80**	**8**	**2.65E-15**
	**GO:0006357**	**regulation of transcription by RNA polymerase II**	**129**	**12.9**	**2.83E-10**
	**GO:0000122**	**negative regulation of transcription by RNA polymerase II**	**78**	**7.8**	**5.24E-10**
	**GO:0045944**	**positive regulation of transcription by RNA polymerase II**	**52**	**5.2**	**0.009144005**
**Cow**	**GO:0006357**	**regulation of transcription by RNA polymerase II**	**124**	**8.940158616**	**6.94E-08**
**Mouse**	**GO:0045944**	**positive regulation of transcription by RNA polymerase II**	**86**	**9.440175631**	**3.18E-12**
	**GO:0045893**	**positive regulation of DNA-templated transcription**	**54**	**5.927552141**	**2.48E-09**
	**GO:0045892**	**negative regulation of DNA-templated transcription**	**51**	**5.598243688**	**2.53E-09**
	GO:0006629	lipid metabolic process	52	5.708013172	4.02E-08
	**GO:0006357**	**regulation of transcription by RNA polymerase II**	**81**	**8.891328211**	**5.19E-07**

	**GO:0006355**	**regulation of DNA-templated transcription**	**74**	**8.122941822**	**6.04E-07**
	**GO:0000122**	**negative regulation of transcription by RNA polymerase II**	**54**	**5.927552141**	**4.47E-05**
	GO:0030154	cell differentiation	49	5.37870472	0.001087541
**Rat**	GO:0008150	biological_process	80	10.70950469	0.016817569
	**GO:0006357**	**regulation of transcription by RNA polymerase II**	**60**	**8.032128514**	**0.021025306**
**Chicken**	ND				
**Frog**	ND				
**Zebrafish**	GO:0035556	intracellular signal transduction	14	5.761316872	0.014442325
**Medaka**	GO:0007165	signal transduction	9	5.294117647	0.052444212
**Elephant shark**	ND				

ND: not detectable. The GO terms associated with DNA binding and transcriptional regulation are indicated in bold letters.
